# Someone at Home: How Kinship Support Influences Aging-in-Place Decisions Among Childless Older Adults in Rural China

**DOI:** 10.1093/geroni/igaa057.1407

**Published:** 2020-12-16

**Authors:** Chen Hongzhou, Vivian Lou, Hongzhou Chen

**Affiliations:** The University of Hong Kong, Hong Kong, China

## Abstract

Background and aim: 80% of childless older adults in rural China choose to ageing-in-place (AIP) rather than moving to residential facilities, regardless the fact that they were financially constrained, scarily supported and some were physically disabled. This research explores the reason why AIP were prevailing preferred. Research design and Method: A qualitative method adopting constructive grounded theory approach was utilized in this research. 20 in-depth interviews were conducted among childless rural residents (aged 60 to 83 years old, 8 of them were over 75 years old) in Yunnan, one of the most economically disadvantaged provinces in China. Data was transcript and coded line-by-line, a in Vivo coding strategy was engaged to capture local language and meanings. Results: A phrase - ‘there’s someone at home’ - was used by rural childless older adults to explain their AIP decision, which demonstrating the role of kinship relations. Three sub-themes were emerged regarding to the phrase: 1) reciprocity, as the support were mutual and predictable; 2) justified conflicts, as older adults and ‘someone’ managing the support relation with subtle conflict; 3) unspoken agreement, as childless older adults being constrained by filial piety when negotiating for further support. All of sub-themes were related with sense of certainty. For participants who were over 75, growing old were “naturally” related with decreased social support. The daily-based kinship support and sense of certainty was particularly important among childless older adults who would like to choose AIP but still questioning the sustainability of self-reliance at an uncertain rural place.

